# Self-Esteem, Self-Monitoring, and Temperamental Traits in Action: Who Is Involved in Humanitarian, Political, and Religious Non-profit Organizations?

**DOI:** 10.3389/fpsyg.2020.573689

**Published:** 2021-01-08

**Authors:** Dorota Kanafa-Chmielewska

**Affiliations:** Institute of Psychology, University of Wrocław, Wrocław, Poland

**Keywords:** self-esteem, self-monitoring, temperamental traits, NPOs, volunteers

## Abstract

Self-esteem, self-monitoring, and temperamental traits are important factors that influence human behavior. The purpose of the present study was to compare groups involved in humanitarian (*n* = 61), political (*n* = 68), and religious (*n* = 54) activities in terms of intergroup differences in self-esteem, self-monitoring, and temperamental traits. There are two research questions that we sought to address: “What are the relationships between self-esteem, self-monitoring, and temperamental traits among those involved in social, religious, and humanitarian aid activities?” and “Do temperamental traits affect self-esteem and self-monitoring among volunteers?” The study was conducted in Poland among adults aged 18 years and older, during meetings of six selected non-profit organizations, consisting of two organizations each in the humanitarian, political, and religious areas. The study used the Polish versions of the Rosenberg Self-esteem Scale, Snyders’ Self-Monitoring Scale, and the EAS Temperament Questionnaire. Volunteers of humanitarian organizations had the lowest self-esteem among all the examined groups. Politicians turned out to be more pragmatic than those involved in religious activities and humanitarian aid. Between the three examined groups, there were statistically significant differences in temperamental activity; the most active are those politically involved, followed by participants involved in humanitarian aid and religious activities. Moreover, volunteers involved in humanitarian aid reported a higher level of temperamental fear than those involved in political and religious activities. Furthermore, there were group differences in their reasons for social involvement. We discuss the potential sources of differences and consequences of outcomes for human resource practice in non-profit organizations.

## Introduction

In modern societies, people increasingly feel the need to engage in activities unrelated to work. Social involvement is valuable because, on its basis, personnel strategies are implemented by non-profit organizations (NPOs) and companies through enriching their activities with corporate social responsibility and employee volunteering. In fact, one can hear about civic behavior in an organization and no one is surprised by the foundations established by some enterprises.

The issue of social involvement in psychology is sometimes considered to be closely related to other phenomena such as attribution of responsibility, control over the course of events, learned helplessness, love, and upbringing (Aronson et al., 2015). Moreover, social involvement is often identified with social activity or political participation, with analysis conducted at all social levels (Verba et al., 1971; Verba and Nie, 1972).

In sociology and political science, social involvement is often considered in relation to civil society, social capital, and trust (Sztompka, 1994, 2006; Putnam, 1995, 2000, 2004). In such a context, the negative aspects of involvement in social bonds are also emphasized (Putnam, 2004). Furthermore, involvement can also mean a lack of objectivity (Elias, 2007, 2011) or interactive coercion (Goffman, 1983).

On the other hand, in psychology, social involvement is defined as behavior directed toward a goal related to the purpose of the secondary group, which is subject to volitional control. Sztompka (2002) defines secondary groups as “those with many members, mostly anonymous, between which formalized and indirect relations occur in highly specialized relations.” Secondary groups include professional groups or social organizations of various types, as well as NPOs. Moreover, social involvement does not result from sanctions (cultural, formal and legal, and institutional), and has a conscious or unconscious instrumental value for the acting individual (Kanafa-Chmielewska, 2016).

In addition, NPOs are:

(a) Formally organized: they possess institutional reality.
(b) Private: they are independent from governments and the public sector.
(c) Self-governing: They have the capacity to control their own activities.
(d) Not-for-profit: they do not redistribute or return any profits generated to owners or shareholders.
(e) Voluntary: they are not compulsory (Anheier, 2000, pp. 1–2, after: Benevene et al., 2011).

The conscious instrumental value of individuals (and volunteers) taking part in a religious organization may be to ensure the salvation of their souls, whereas the unconscious value may be to satisfy the need for affiliation. Dedicating time to a humanitarian organization without any remuneration is consistent with the purpose of that organization, which is to recruit more volunteers. In such a case, the conscious instrumental value may be to help others, while the unconscious instrumental value may be to enable individuals to perceive themselves as good or generous. On the other hand, membership in a political association, as compared to humanitarian organizations, is linked to the purpose of the association—having as many members as possible to increase the chances of achieving specific political goals. In such a case, the conscious instrumental value may be the opportunity to meet politicians, whereas the unconscious instrumental value may be the desire to gain power and popularity (Kanafa-Chmielewska, 2016).

Despite its importance, the lack of psychology research on this aspect is noticeable; thus, inquiries regarding the available conditions for social involvement should be the focus of research. There is a need to distinguish the subjective determinants of social involvement. In this study, in choosing the variables for the research model, attention was paid to dispositional conditions that determine contact with other people in social situations that are of a collaborative nature. Self-esteem and self-monitoring were chosen as variables because of their usefulness in forecasting the behavior of individuals in social situations. *Self-esteem* is an attitude toward the self, which consists of emotions associated with cognitive judgment (Rosenberg, 1965). On the other hand, *self-monitoring* (observational self-control of behavior, see Snyder, 1974; Snyder et al., 1985) or *pragmaticism* is the degree to which people want and are able to manage their own expressive (i.e., verbal and non-verbal) behaviors, and the way they present themselves to others depending on the situation, which determines to what extent the individual is a social chameleon (Wojciszke, 2002).

Due to the temperamental conditions of self-esteem and self-monitoring, temperament was also taken into account. *Temperament* refers to a group of personality traits that appears at an early stage of human development; these traits have two special properties, i.e., they are genetically determined and appear in the first year of life, which distinguishes them from other personality traits, and they are both inherited and acquired. These personality traits include activity, sociability, fear, dissatisfaction, and anger (Goldsmith et al., 1987, pp. 508).

The inspiration for undertaking this research is the growing importance of the associations and foundations that exist in Western countries and in Poland. This is because the collapse of the communist system opened up the space to satisfy the Polish people’s need for political, social, economic, and cultural involvement. Thus, the rationale for this study is the realization of a perspective based on the intellectual capital (IC) framework, which is the primary theoretical approach for studying intangible assets in organizations (Buonomo et al., 2020).

The goal of the study is to recognize the differences and similarities among volunteers who devote their time to politics, religious communities, and humanitarian activities. We sought to answer two questions: “What are the relationships between self-esteem, self-monitoring, and temperamental traits among those involved in social, religious, and humanitarian aid activities?” and “Do temperamental traits affect self-esteem and self-monitoring among volunteers?”

The present research contributes to the literature by indicating the characteristics of people working in NPOs, which enables them to be grouped according to their preferred attributes. This can help NPOs in the achievement of their objectives to gain a better understanding of their active and potential volunteers.

## Materials and Methods

### Participants and Procedure

The study focused on three types of social involvement: political, religious, and humanitarian aid. Participants were adults aged 18 and above, who provided data during meetings of selected non-profit organizations. Only volunteers—that is, people working for the organization without remuneration—were asked to complete the paper-and-pencil questionnaires, and there was no time limit. Participants received no financial compensation for taking part in the study. Data were collected following the American Psychological Association’s (APA) general principles and ethical research standards. The Institute of Psychology, University of Wrocław Research Ethics Committee approved the research procedure and methods of this study.

#### Politically Involved

Regarding political involvement, we examined the members of youth organizations affiliated with political parties, because access to party activists was limited. A total of 68 people were examined, from which 37 belonged to the youth organization of the right-wing party (RWP), while 31 were from the youth organization of the center-oriented party (COP). In both groups, women were a minority (i.e., nine participants from RWP and 11 from COP were women). For RWP, the average age was 24 (range = 18–30, *SD* = 3), while for COP, the average age was 22 (range = 18–29 years, *SD* = 3). The average period of political involvement of the members of both organizations was 4 years.

#### Religiously Involved

Social involvement in matters of the religious community was examined among Protestants. The Poles are mostly Roman Catholics (CBOS, 2018), but rather not perform activities undertaken for the benefit of the community (e.g., planning joint trips, conducting cooking courses, or learning foreign languages), other than participation in the mass. This paper considered “social involvement in matters of a religious community” to be the same as “religious involvement.” For this reason, Protestants were selected for this study as they are more strongly involved in the social matters of their religious community than Roman-Catholics.

A total of 54 Protestants were examined. Among them, 22 were considered New Protestants (NP), or those who converted from Roman Catholicism and joined the Protestant community as adults; hence, they were isolated in some of the analyses. Adults aged 18 years and above were allowed to participate in the study. Among the Protestants (19 men and 13 women), most were between 41 and 50 years old (28%; *M*age = 35 years, range = 19–62, *SD* = 13), while for the NP (nine men and 13 women), the ages ranged between 31 and 40 years (64%; *M*age = 39 years, range 30–71, *SD* = 10). Notably, there were no people under the age of 24 and between 51 and 60 years among the NP. The average period of social involvement in matters of the religious community was over 17 years for Protestants and almost 13 years for NP.

#### Involved in Humanitarian Aid

The study of social involvement in humanitarian aid was conducted in two organizations. The first was the local foundation (LF), an organization that helps the terminally ill and their families, and operates in several large cities in Poland. The second was the global foundation (GF), which is a large Polish organization that operates worldwide and provides assistance in various types of crises (e.g., education, shaping humanitarian attitudes, and building a modern culture of assistance). Both organizations differ in their forms of providing assistance and areas of activity. A total of 62 people were investigated; 31 for LF (*M*age = 27 years, range = 18–59, *SD* = 10; 25 were women) and 31 for GF (*M*age = 32 years, range = 21–67, *SD* = 11; 28 were women). The average period of social involvement was almost 2 years for LF and nearly 4 years for GF.

### Instruments

#### Self-Esteem

Self-esteem was assessed using the Polish adaptation of Rosenberg’s Self-esteem Scale (RSES, Rosenberg, 1965). It consists of ten items that measure global self-worth using positive and negative feelings about the self, with higher scores indicating greater self-esteem. The answer scale ranged from 1 (*strongly agree*) to 4 (*strongly disagree*). Several studies have shown the questionnaire’s psychometric properties, supporting its use in different languages (Rosenberg, 1965; Silber and Tippett, 1965; Kernis et al., 1991; Hagborg, 1993; Dzwonkowska et al., 2008). In the present study, Cronbach’s alpha was 0.84.

#### Self-Monitoring

Self-monitoring was assessed using the Polish adaptation of Snyder’s (1974) Pragmaticism Scale (Wojciszke, 1984). It measures the extent to which people consider themselves as *pragmatists*, or those who adapt their behavior to various situations and choose the role they will play based on the circumstances in which they have to function, or *principalists*—those who want to remain guided by their dispositions and internal states in every situation. This one-dimensional scale consists of 29 items answered with “true” or “false,” with higher scores indicating greater self-monitoring levels. In the present study, Cronbach’s alpha was 0.76.

#### Temperament

Temperament traits were measured using the Polish version of the EAS Temperament Questionnaire for adults (EAS-D) by A. H. Buss and R. Plomin (Oniszczenko, 1997). This questionnaire was designed to investigate five temperament traits (i.e., fear, anger, dissatisfaction, activity, and sociability). It comprises 20 items (i.e., four items for each of the five subscales) and is scored using a five-point Likert-scale ranging from 1 (*definitely not*) to 5 (*definitely yes*), with higher scores indicating greater trait levels. Its reliability, measured by Cronbach’s alpha coefficients, was 0.74 for dissatisfaction, 0.7 for fear, 0.5 for anger, 0.6 for activity, and 0.45 for sociability; the reliability of three out of five temperament subscales is below 0.7, which is considered appropriate for the research method.

Dissatisfaction (emotionality-distress), called undifferentiated emotionality, is the tendency to react easily and strongly with anxiety. One end of emotionality is defined by being unresponsive, while the other is defined by intense, unpredictable, or uncontrolled reactions, such as crying, screaming, or being in a bad mood. Fear (emotionality-fear) is distinguished from dissatisfaction in the second or third month of life. The indicators of fear are the tendency to avoid aversive stimuli and the tendency to run away from a threat, along with fear, crying, or screaming. Anger (emotionality-anger) appears around 6 months of age, after dissatisfaction that may manifest from birth and fear that stands out around 2 or 3 months of age. Anger is caused by frustrating or irritating stimuli that result in mimic, motor, and cognitive responses. Activity refers to only physical exertion, including motor activities of the head, limbs, and torso. In the theoretical context, the scope of the described feature does not include mental effort or cognitive processes. An indicator of sociability is the desire to be with other people. Loneliness causes frustration in people who have a high level of this trait. Sociability manifests itself in striving to make friends and maintaining long-term contacts that provide positive reinforcements (Oniszczenko, 1997).

The elements that make up the activity can be distinguished when the child begins to control their motor activity. They include pace and vigor, and, less important endurance and motivation. Pace is the speed of an action, such as speaking or walking. Vigor, on the other hand, is related to the strength or intensity of reactions such as laughing, shouting, speaking, or treading. In the described theory, activity is the only trait that, according to Buss and Plomin (1984), relates to the style and not the content of the behavior.

Social rewards include being together and undertaking cooperative activities such as talking, playing, or eating. Sociability can be measured, for example, by the frequency of contact initiation, the number of connections, or the time spent in the company of other people. It is also possible to check how individuals react to social isolation (Buss and Plomin, 1986).

#### Reasons for Involvement

Reasons for involvement were measured using a tool consisting of eight statements prepared by the author of the article: (1) I am in an organization/community because it helps me realize my ambitions; (2) I am in an organization/community because I meet my friends here; (3) I am in an organization/community because I generally like to act; (4) I am in an organization/community because I want to do something for others; (5) I am in an organization/community to continue my family tradition; (6) I am in an organization/community because it allows me to put into practice the values that I profess; (7) I am in an organization/community out of habit; and 8) I am in an organization/community due to a sense of duty. The answer scale ranged from 1 (*strongly disagree*) to 5 (*strongly agree*).

Realizing ambitions refers to gaining experience and climbing the organizational career ladder. Working in NPOs is an opportunity to meet new people, make friends, and expand social networks. “I like to act” is a statement related to doing something, being engaged, and responding to stimuli. “I like to do something for others” is a declaration of altruism. Some people who continue family traditions, such as with siblings or parents, are active in NPOs. “I put into practice the values that I profess” means that individuals feel the need to turn their beliefs into behaviors. Being in an organization out of habit means that someone does not want to change something, even if that thing happened accidentally (for example, accidentally arriving to a meeting and staying). It does not refer to a high, conscious motivation to act in NPOs. A sense of duty stems from values that indicate that something should be done.

The reasons for the activity were initially formulated in conversations with people operating in the non-profit sector, as the results of focus group interviews conducted at the University of Wrocław among postgraduate students, and from the literature on the subject (see Verba et al., 1971; Verba and Nie, 1972; Himmelfarb, 1975).

### Statistical Analysis

Analyses were conducted using SPSS software (version 25.0; IBM) to compute for descriptive statistics, internal consistencies, and bivariate correlations. We used ANOVA to identify the differences and similarities in self-esteem, self-monitoring, and temperamental traits among the three groups. Additionally, linear stepwise regression was carried out to determine the effect of temperamental traits on self-esteem and self-monitoring.

## Results

Table 1 presents the descriptive statistics for the total sample (*N* = 184), Cronbach’s alpha values, and correlations among the considered variables.

**TABLE 1:** Descriptive statistics for total sample, Cronbach’s alpha values, and correlations among considered study variables (*n* = 184).

	***M***	**SD**	**α**	**1**	**2**	**3**	**4**	**5**	**6**	**7**	**8**	**9**	**10**	**11**	**12**	**13**	**14**	**15**	**16**
SE	30.86	4.377	0.84	1															
SM	17.39	4.790	0.76	0.152*	1														
DIS	9.83	3.214	0.74	−0.567**	−0.089	1													
FE	9.79	3.328	0.70	−0.596**	−0.206**	0.717**	1												
AN	11.42	2.941	0.50	−0.190**	−0.065	0.559**	0.358**	1											
AC	14.26	2.885	0.60	0.196**	0.272**	−0.180*	−0.217**	0.082	1										
SO	14.09	2.609	0.45	0.430**	0.216**	−0.386**	−0.289**	−0.162*	0.333**	1									
Ambition	3.52	1.135	n/a	0.169*	0.345**	−0.098	−0.232**	−0.010	0.307**	0.194**	1								
Friends	3.43	1.089	n/a	0.120	0.255**	−0.112	−0.154*	−0.103	0.101	0.251**	0.355**	1							
Acting	3.93	1.014	n/a	0.201**	0.257**	−0.145	−0.118	0.030	0.343**	0.259**	0.588**	0.374**	1						
Altruism	4.05	0.851	n/a	0.088	0.128	−0.069	0.042	−0.087	0.202**	0.141	0.369**	0.272**	0.650**	1					
Tradition	2.09	1.001	n/a	0.010	0.118	−0.019	−0.071	0.042	0.058	−0.043	0.135	0.118	0.082	0.027	1				
Values	4.28	0.689	n/a	0.274**	0.027	−0.104	−0.086	−0.020	0.073	0.235**	0.087	0.101	0.146**	0.238**	−0.045	1			
Habit	1.95	0.841	n/a	−0.113	0.017	0.124	0.056	0.042	−0.046	−0.013	0.001	0.229**	−0.056	−0.103	0.175*	−0.153*	1		
Duty	2.60	1.155	n/a	−0.077	0.127	0.037	−0.022	0.099	0.056	−0.035	0.250**	0.151*	0.247**	0.098	0.216**	0.002	0.343**	1	
Age	29.25	10.669	n/a	−0.020	−0.324**	−0.025	0.018	−0.053	−0.285**	−0.070	−0.279**	−0.192**	−0.422**	−0.266**	−0.072	0.078	−0.168*	−0.207**	1

**p < 0.01 (two-tailed), *p < 0.05 (two-tailed); temperamental traits: SE, self-esteem; SM, self-monitoring; DIS, dissatisfaction; FE, fear; AN, anger; AC, activity; SO, sociability; reasons for involvement: ambition, friends, acting, altruism, family tradition, values, and sense of duty.

Self-esteem correlates with all temperament traits; we found a negative correlation with dissatisfaction and fear, and highly positive correlation with sociability. The higher the self-esteem, the more likely the motives (i.e., ambition, willingness to act, and values) are related to social activity. Overall, self-esteem was low and positively correlated with pragmaticism.

On the other hand, pragmaticism was low and correlated negatively with fear and positively with activity and sociability. The motives for social activity include ambition, willingness to meet friends, and willingness to act. The older the individuals, the less prone they are to self-monitoring.

### Self-Esteem

ANOVA using the Scheffé’s *post hoc* criterion for significance [*F*(2,181) = 9.353; *p* < 0.001] revealed that volunteers involved in humanitarian aid had lower self-esteem (*M* = 29.03, *SD* = 4.29) than those in political (*M* = 32.10, *SD* = 4.22) and religious communities (*M* = 31.41, *SD* = 4.03).

### Self-Monitoring

ANOVA using the Scheffé’s *post hoc* criterion for significance revealed that the highest scores in self-monitoring were among young politicians [*M* = 19.84, *SD* = 4.08; *F*(2,181) = 9.353; *p* < 0.001]. Volunteers involved in religious communities (*M* = 15.61, *SD* = 4.69) and humanitarian aid (*M* = 16.24, *SD* = 4.54) did not differ in their level of pragmaticism.

### Temperament

ANOVA using the Scheffé’s *post hoc* criterion for significance revealed differences in two out of five temperamental traits between the studied groups. The highest level of fear [*F*(2,181) = 7.539; *p* < 0.001] was found in those involved in humanitarian aid (*M* = 10.97, *SD* = 3.43), followed by those involved in politics (*M* = 8.78, *SD* = 3.10); those involved in religious activities were not statistically different from the other two groups (*M* = 9.70, *SD* = 3.09). The highest activity [F(2,181) = 7.707; *p* < 0.001] was demonstrated by politically involved participants (*M* = 15.24, *SD* = 2.82) compared to those involved in religious (*M* = 13.28, *SD* = 2.43) and humanitarian aid (*M* = 14.05, *SD* = 3.02).

### Reasons for Involvement

Table 2 presents the descriptive statistics of the reasons for involvement of the total sample (*n* = 184). Young politicians (*n* = 68) reported the most important reasons for involvement: willingness to act (*M* = 4.43), putting values into practice (*M* = 4.26), the pleasure of doing something for others (*M* = 4.16), and implementation of ambition (*M* = 4.10). On the other hand, Protestants (*N* = 54) indicated the following reasons for their activity: putting values into practice (*M* = 4.44), willingness to do something for others (*M* = 3.63), meeting friends (*M* = 3.35), and willingness to act (*M* = 3.09). In contrast, volunteers of humanitarian organizations (*N* = 62) declared that they are active because they like to do something for others (*M* = 4.29), put values into practice (*M* = 4.15), like to act (*M* = 4.11), and realize their ambitions (*M* = 3.53).

**TABLE 2:** Descriptive statistics of reasons for involvement for total sample (*n* = 184).

		**Ambition**	**Friends**	**Acting**	**Altruism**	**Tradition**	**Values**	**Habit**	**Duty**
Politics	*M*	4.10	3.90	4.43	4.16	2.26	4.26	2.07	3.15
	SD	0.78	0.79	0.65	0.70	1.13	0.61	0.87	1.16
Religion	*M*	2.78	3.35	3.09	3.63	2.09	4.44	1.81	2.04
	SD	1.14	1.15	1.07	1.10	1.03	0.69	0.75	0.91
Humanitarian aid	*M*	2.78	3.35	3.09	3.63	2.09	4.44	1.81	2.04
	SD	1.14	1.15	1.07	1.10	1.03	0.69	0.75	0.91

*Reasons for involvement: ambition, friends, acting, altruism, family tradition, values, habit and sense of duty.*

*Post hoc* analyses using the Scheffé’s *post hoc* criterion for significance indicated that there were five motives that differentiated the social involvement of the three groups. First, the desire to realize ambition [*F*(2,181) = 26.14; *p* < 0.001] was the most important for young politicians, followed by those involved in humanitarian aid and religious communities (*M*_PI_ > *M*_HAI_ > *M*_RI_; *p* < 0.05).

Second, meetings with friends [*F*(2,181) = 12.64; *p* < 0.001] was more important for politicians than people from the other two groups (*M*_PI_ > *M*_HAI_ and *M*_PI_ > *M*_RI_; *p* < 0.05) who reported no differences in this regard.

Third, willingness to act [*F*(2,181) = 39.06; *p* < 0.001] was low in the case of people involved in religion compared to those involved in politics or humanitarian aid (*M*_PI_ > *M*_RI_ and *M*_HAI_ > *M*_RI_; *p* < 0.05), who reported no differences in this area.

Fourth, satisfaction of doing something for others [*F*(2,181) = 10.66; *p* < 0.001] was low in the case of people involved in religion compared to those involved in politics or humanitarian aid (*M*_PI_ > *M*_RI_ and *M*_HAI_ > *M*_RI_; *p* < 0.05), who reported no differences in this area.

Fifth, sense of duty [*F*(2,181) = 16.73; *p* < 0.001] was more important for politicians than the other two groups. However, the results of Tukey’s and Scheffé’s *post hoc* tests did not provide data that would indicate differences between Protestants and volunteers of humanitarian organizations (*M*_PI_ > *M*_RI_ and *M*_PI_ > *M*_HAI_; *p* < 0.05).

The model of the association between temperamental traits and self-esteem, which was obtained using linear stepwise regression, is presented below (Table 3). The model of the relationship between temperamental traits and self-monitoring was also checked; however, it explained only 9% (*R*^2^ = 0.087) of the dependent variable (i.e., self-monitoring). Because of this, it will not be presented.

**TABLE 3:** Model summary (*n* = 184).

**Model**	**R**	**R-square**	**Adjusted R-square**	**Std. error of the estimation**
1	0.596^a^	0.355	0.352	3.525
2	0.654^b^	0.428	0.421	3.329
3	0.668^c^	0.446	0.436	3.286
4	0.678^d^	0.459	0.447	3.254

*^*a*^Dependent variable: self-esteem. ^*b*^Predictor: (constant), fear. ^*c*^Predictors: (constants), fear, sociability. ^*d*^Predictors: (constants), fear, sociability, dissatisfaction. ^*e*^Predictors: (constants), fear, sociability, dissatisfaction, anger.*

The model with five predictors explained 45% (*R*^2^ = 0.447) of the dependent variable [i.e., self-esteem; *F*(4,179) = 38.04; *p* < 0.001] (Table 3).

Values of standardized coefficients for fear (β = −0.371, *p* < 0.001), sociability (β = 0.234, *p* < 0.001), dissatisfaction (β = −0.291, *p* < 0.01), and anger (β = 0.143, *p* < 0.05) are shown in Table 4.

**TABLE 4:** Model coefficients (*n* = 184).

**Model**		**Unstandardized coefficients**		**Standardized coefficients**	***t***	**Sig.**
				
		**B**	**Std. Error**	**Beta**		
1	(Constant)	38.535	0.809		47.623	0.000
	Fear	−0.784	0.078	−0.596	−10.010	0.000
2	(Constant)	30.827	1.780		17.320	0.000
	Fear	−0.677	0.077	−0.514	−8.758	0.000
	Sociability	0.473	0.099	0.282	4.795	0.000
3	(Constant)	32.716	1.924		17.001	0.000
	Fear	−0.504	0.105	−0.383	−4.813	0.000
	Sociability	0.407	0.101	0.243	4.034	0.000
	Dissatisfaction	−0.271	0.113	−0.199	−2.405	0.017
4	(Constant)	31.574	1.978		15.960	0.000
	Fear	−0.488	0.104	−0.371	−4.691	0.000
	Sociability	0.392	0.100	0.234	3.914	0.000
	Dissatisfaction	−0.396	0.126	−0.291	−3.148	0.002
	Anger	0.213	0.099	0.143	2.145	0.033

*For all models the dependent variable is self-esteem.*

The regression equation is as follows:

$$\overset{´}{\text{Y}}=31.57-0.49\times X_{\mathrm{fea}}+0.39\times X_{\mathrm{soc}}-0.40\times X_{\mathrm{diss}}$$

$$\;+0.21\times X_{\mathrm{ang}}$$

On this basis, it can be concluded that the higher the self-esteem, the more sociable the people are, and the more likely they are to display anger in defense of their goals. At the same time, the higher self-esteem, the lower the levels of fear and dissatisfaction.

## Discussion

Participation in NPOs is recognized as the foundation of a civil society, which is essential at both the local and international levels (e.g., the establishment of the European Commission, 2020). NPOs have become allies of both the citizens and governments in the fight against social problems, especially poverty and social exclusion (ec.europa.eu, 10.06.2020)^1^. In connection with the growing demand for services in the so-called third sector, there is a need to better understand the personnel who work in these organizations. As there is limited information regarding the characteristics of volunteers involved in third-sector organizations, the present study may help to fill this gap.

Surprisingly, volunteers from humanitarian aid organizations had the lowest self-esteem among all the groups in this study. In effect, this result brings to mind the well-known anecdote about Abraham Lincoln with regard to helping as a human behavior. As the story goes, while traveling with a friend through wetlands, they discussed the motives of human actions. Lincoln claimed that in doing both good and evil, everyone is motivated by selfishness. Lincoln’s friend barely managed to dispute his assertion when they heard a terrifying noise and found piglets drowning in a swamp. Immediately, and ignoring the fact that he would damage his clothes, Lincoln helped the animals. After the rescue, Lincoln’s friend asked how this situation relates to his beliefs regarding the motivation of people. Lincoln replied: “That was the very essence of selfishness. I would have had no peace of mind all day had I gone on and left that suffering old sow worrying over those pigs. I did it to get peace of mind, do you not see?” Perhaps doing something for others is actually acting on behalf of self-esteem (cf. self-esteem as sociometer theory, Leary and Baumeister, 2000). Thus, the issue regarding the motivation of people in helping others requires further scientific exploration.

Participants who were politically involved reported the highest score in self-monitoring. This result is in line with the intuition that comes from observing everyday life. There is probably a politician in each country who, in the eyes of citizens, has changed their political beliefs from left to right or vice versa. Even if the changes are insignificant, political flexibility and being a situational chameleon are necessary for surviving in a political environment. Hence, the chameleon-like tendencies attributed stereotypically to politicians (Schuetz, 2006) were confirmed.

The outcomes on temperamental traits remain with regard to self-esteem and self-monitoring. The highest level of fear was observed among those involved in humanitarian aid, which may indicate a threat to their self-esteem. Coping with low self-esteem among humanitarians can be explained by the theory of fear management (Greenberg et al., 1997), which states that doing something for others is a method of fear management. On the other hand, the highest activity was noticed among politically involved participants, which may be attributed to their observational self-control of the environment.

The proposed model of the relationship between temperamental traits and self-esteem indicated that higher self-esteem leads to more sociability among people and a tendency to display anger in defense of their goals. Higher self-esteem is accompanied by lower fear and dissatisfaction. On the other hand, self-monitoring turned out to be slightly dependent on temperamental traits. Thus, some other internal or situational factors are responsible for its expression.

The above findings are better understood when differences in motives for social involvement are considered. A desire to realize ambitions, meeting friends for both recreational and pragmatic (e.g., making alliances) reasons, and possessing a sense of duty are crucial for young politicians. However, the question regarding to whom or what they feel their greatest duty toward (e.g., for themselves, their country, or others) may indicate the direction of further research. On the contrary, willingness to act is of the utmost importance for young politicians and humanitarian aid volunteers, which may be interpreted as a compulsion to act for a career or self-esteem. An unexpected low score on satisfaction with doing something for others was reported by those religiously involved, in contrast to the other two groups.

This research is not free from limitations. In this study, we used self-report questionnaires for data collection in a cross-sectional study design. However, the participants were among the most active volunteers, which was confirmed by those performing managerial functions in the respective examined organizations. Additionally, the sample size should be increased to promote the generalizability of these results.

Despite these limitations, the present study makes a considerable contribution in exploring the chosen characteristics (i.e., self-esteem, self-monitoring, and temperamental traits) of volunteers involved in politics, religious communities, and humanitarian aid as far as we are concerned, it is the first study that address this issue. The results shed light on motives for involvement, which could be used in recruiting volunteers, motivating them, and helping them deal with their workloads. However, due to the specificity of the sectors involved in this study, there are probably fewer candidates than the unpaid positions that need to be filled.

Future research should focus on exploring the psychological characteristics of volunteers in various non-profit organizations. The second direction of future research could be the analysis of the effectiveness of unpaid work and its top performers.

## Data Availability Statement

The raw data supporting the conclusions of this article will be made available by the authors, without undue reservation.

## Ethics Statement

Ethical review and approval was not required for the study on human participants in accordance with the Local Legislation and Institutional Requirements. Written informed consent for participation was not required for this study in accordance with the National Legislation and the Institutional Requirements.

## Author Contributions

DK-C developed the study design and survey, performed the data collection and analysis, and contributed to writing the manuscript.

## Conflict of Interest

The author declares that the research was conducted in the absence of any commercial or financial relationships that could be construed as a potential conflict of interest.

## Footnotes

1 ec.europa.eu
